# 

**Published:** 1920-06

**Authors:** 


JUNE, 1920.

*Vol. XXXVII.* *No.* 139.

Library of Congress
JUL 27 1920
Smithsonian Deposit

### THE BRISTOL MEDICO-CHIRURGICAL JOURNAL

*PUBLISHED UNDER THE AUSPICES OF*

*THE BRISTOL MEDICO-CHIRURGICAL SOCIETY.*

*Editor:* P. WATSON-WILLIAMS, M.D.,

WITH WHOM ARE ASSOCIATED

J. LACY FIRTH, M.S., M.D., JAMES SWAIN, C.B., C.B.E., M.S., M.D., CAREY COOMBS, M.D.

J. A. NIXON, C.M.G., M.D., *Assistant Editor.*

*Editorial Secretary:* J. M. FORTESCUE-BRICKDALE, M.A., M.D.

BRISTOL: J. W. ARROWSMITH LTD.

LONDON: SIMPKIN, MARSHALL, HAMILTON, KENT & CO. LTD.

*Price Three Shillings net.*

